# Preparation and Performance Evaluation of a Supramolecular Polymer Gel-Based Temporary Plugging Agent for Heavy Oil Reservoir

**DOI:** 10.3390/gels10080536

**Published:** 2024-08-19

**Authors:** Cheng Niu, Sheng Fan, Xiuping Chen, Zhong He, Liyao Dai, Zhibo Wen, Meichun Li

**Affiliations:** 1 Petroleum Engineering Technology Institute of Northwest Petroleum Branch, SINOPEC, Urumqi 830011, Chinachenxp3776.xbsj@sinopec.com (X.C.);; 2 Key Laboratory of Enhanced Oil Recovery in Carbonate Fractured-Vuggy Reservoirs, SINOPEC, Urumqi 830011, China; 3 School of Petroleum Engineering, China University of Petroleum (East China), Qingdao 266580, China; b22020047@s.upc.edu.cn (L.D.);; 4 State Key Laboratory of Deep Oil and Gas, China University of Petroleum (East China), Qingdao 266580, China

**Keywords:** fractured formation, gel, heavy oil reservoir, supramolecular polymer, temporary plugging material

## Abstract

When encountering heavy oil reservoirs during drilling, due to the change in pressure difference inside the well, heavy oil will invade the drilling fluid, and drilling fluid will spill into the reservoir along the formation fractures, affecting the drilling process. A supramolecular polymer gel-based temporary plugging agent was prepared using acrylamide (AM), butyl acrylate (BA), and styrene (ST) as reacting monomers, N, N-methylenebisacrylamide (MBA) as a crosslinking agent, ammonium persulfate (APS) as an initiator, and poly(vinyl alcohol) (PVA) as a non-covalent component. A supermolecular polymer gel with a temperature tolerance of 120 °C and acid solubility of 90% was developed. The experimental results demonstrated that a mechanically robust, thermally stable supramolecular polymer gel was successfully synthesized through the copolymerization of AM, BA, and ST, as well as the in situ formation hydrogen bonding between poly (AM-co-BA-co-ST) and PVA, leading to a three-dimensional entangled structure. The gel-forming solution possessed excellent gelling performance even in the presence of a high content of salt and heavy oil, demonstrating superior resistance to salt and heavy oil under harsh reservoir conditions. High-temperature and high-pressure plugging displacement experiments proved that the supramolecular polymer gel exhibited high pressure-bearing capacity, and the blocking strength reached 5.96 MPa in a wedge-shaped fracture with a length of 30 cm. Furthermore, the dissolution rate of the supramolecular polymer gel was as high as 96.2% at 120 °C for 48 h under a 15% HCl solution condition.

## 1. Introduction

In the field of petroleum exploration and development, the extraction of heavy oil resources has always been a challenging subject [1,2,3]. Heavy oil is defined as high-viscosity, high-density crude oil. During the development process, when drilling heavy oil reservoirs, the change in pressure difference in the well can cause heavy oil to invade the wellbore along the fractures, affecting the well productivity, the performance of drilling fluid as well as the drilling process [4,5,6]. First, heavy oil has a high viscosity, making it more difficult to flow through the wellbore, which significantly reduces the well’s productivity. Second, the drilling fluids can be polluted by heavy oil, leading to changes in their rheology and filtration performance. Third, when the heavy oil is excessively invaded, it can lead to complete blockages of the wellbore, resulting in pipe sticking, which necessitates frequent wellbore cleaning operations and increases maintenance costs [7,8]. Therefore, it is of great significance to develop an efficient strategy to prevent heavy oil intrusion from the reservoir into the wellbore [9,10].

Regarding the problem of heavy oil invading drilling fluid, in the early stage of heavy oil invading drilling fluid, the method of increasing density can be used to balance the formation pressure and prevent further invasion of heavy oil into the wellbore. However, on-site application results have shown that increasing the density of drilling fluid cannot completely control the invasion of heavy oil. When the invasion of heavy oil is large, temporary sealing is mainly used domestically and internationally to temporarily block the formation fractures.

Temporary plugging is a promising approach to address this issue. It is a typical technique used in oil and gas drilling and well operations to temporarily block or seal fractures of the wellbore [11,12]. This method helps avoid the intrusion of heavy oil from the reservoir into the wellbore along the fracture during the drilling process. Temporary plugging materials are key to the success of this technique, as they should not only be mechanically robust to resist the intrusion of heavy oil but also be easily removed or dissolved after the plugging purpose is served for superior reservoir protection. According to their type, temporary plugging materials are mainly divided into particle type, fiber type, and liquid type [11,13,14,15]. The particle-type temporary plugging material is injected into the reservoir through the fluid, and then a temporary plugging effect is formed through the accumulation of particles. Zhu et al. [16]. have prepared a degradable preformed particle gel with AM and AMPS as the main body. Gel particles are suitable for reservoirs below 40 °C, and the plugging strength of cores can reach 20 MPa. The gel particles can be expanded to fill the formation fractures. However, gel particles may lack selectivity when plugging formation fractures; that is, they cannot accurately control the specific location and scope of plugging. Li et al. [17] used polyethylene wax particles as the main raw material to prepare an oil-soluble polyethylene wax particle temporary plugging agent. This temporary plugging agent solved the problems of low strength, low permeability recovery, and narrow application range of oil-soluble temporary plugging agents. The fiber-type temporary plugging material is injected into the reservoir through the softening effect of the fluid. The fiber can first flow into the high-permeability layers and fractures, then form a bridging effect. With the increase in the injected fluid, the fiber gradually winds to form a fiber layer, leading to a robust plugging effect. Ma et al. [18] temporarily blocked the fractures using three kinds of fibers and found that the higher the fiber mass fraction, the worse the dispersion. Fiber sealing for 1 mm fractures can achieve a maximum compressive strength of 5 MPa. However, the fiber-type temporary plugging agent has limited pressure-bearing capacity and poor mechanical properties. The liquid-type temporary plugging material is in the liquid state before reaction, and it can form a plugging layer under the action of crosslinking within the fractures in the reservoir. Polymer gel-based temporary plugging agents is in the liquid state before reaction, and it can form a plugging layer under the action of crosslinking within the fractures in the reservoir. At present, phenolic resin, epoxy resin, and acrylamide-based gel materials are mostly used, which can be filled according to the change in cracks and have high plugging strength [19,20,21,22]. Zhang et al. [23] used sodium aldehyde alginate (ASA) and amino gelatin (AG) to form a primary network through Schiff base reaction and added polylactic acid (PLA) fiber to form a three-dimensional network. Then, Ca^2+^ was used as an ionic crosslinking agent to prepare a series of new temporary plugging agents. The results showed that the strength and heat resistance of the hydrogel was enhanced by the combination of fiber and crosslinker. The system effectively sealed a 1 mm fracture at 120 °C and 5 MPa. The current research mainly focuses on the development of high-temperature-resistant polymers, the preparation of crosslinking agents and the filling of inert materials in gel. However, these cured materials have disadvantages such as high cost and difficulty in removing blockages after curing.

Supramolecular chemistry provides a new research direction for the development of temporary blocking materials. Supramolecular gels are multi-level assemblies in which there are many non-covalent interactions (e.g., hydrogen bonding, coordination, host–guest interaction, etc.) [19,24,25,26,27,28]. Compared with high-temperature-resistant polymers, supramolecular polymer gel can achieve certain stimulus responses. Du et al. prepared a thermally responsive dynamic temporary sealing agent using β—cyclodextrin, methyl cellulose, and octanol. The material is in a sol state under low-temperature conditions. With the increase in temperature, the fluid gradually changes into a gel of sufficient strength, which can block the fracture at 90 °C, and the plugging strength can reach 6.8 MPa. When the temperature continues to rise to 110 °C, the gel turns into sol. This kind of heat-induced gel system with β—cyclodextrin as the main body has been widely studied [29,30,31,32]. It is a low-viscosity fluid at low temperatures, retrogel under the stimulation of high temperatures, and will automatically degrade into a sol state at continuous high temperatures. However, this system overly relies on temperature changes and requires precise control of the fracture temperature in the sealed formation. Yang et al. [33] prepared a supramolecular gel based on hydrophobic association and hydrogen bonding by using acrylamide and methyl propylene octadecyl ester. The tensile stress of gel can reach 0.703 MPa, and the plugging strength for 0.5 mm fractures can reach 6.7 MPa. At 135 °C, the gel still has suitable gel strength. Such supramolecular gel can maintain excellent plugging performance under high-temperature conditions, but high degradability is still challenging.

In this paper, the temperature distribution of heavy oil reservoirs in the Tahe area (120 °C) is considered, and the limitations of current temporary plugging agents are addressed. A supramolecular polymer gel with superior plugging effect and degradation performance is prepared through in situ free radical polymerization. This supramolecular polymer gel consists of poly (acrylamide-co-butyl acrylate-co-styrene) (PABS) and polyvinyl alcohol (PVA), in which the covalent and non-covalent interleaving network co-exist, leading to high mechanical strength and acid degradability. In order to ensure the temperature resistance performance in high-temperature reservoirs, we use the rigid segments of ST to improve the temperature resistance of the system and the flexible monomers of BA to enhance the toughness of the system. MBA molecules contain two double bonds, which enable them to connect with multiple polymer chains during free radical polymerization, forming a three-dimensional covalent network structure. In addition, the amide and ester groups in a molecular chain can form strong hydrogen bonds with hydroxyl groups in PVA, generating the non-covalent bonding within the gel, which can be easily broken upon the addition of acid, leading to superior acid degradability. The gel system is not only less affected by temperature in the formation but also has a large number of non-covalent effects, which can be added at any time for plugging removal. The subsequent degradation time of the gel can be adjusted according to the concentration of acid.

In view of the limitations of current temporary plugging agents, a supramolecular polymer gel with superior plugging effect and degradation performance is prepared through in situ free radical polymerization. This supramolecular polymer gel consists of poly (acrylamide-co-butyl acrylate-co-styrene) (PABS) and polyvinyl alcohol (PVA), in which the covalent and non-covalent interleaving network co-exist, leading to high mechanical strength and acid degradability. Particularly, the monomers of acrylamide, butyl acrylate, and styrene were polymerized using ammonium persulfate (APS) as the initiator, creating a robust covalent bond. The presence of styrene further enhanced the rigid polymer chain, leading to high mechanical strength. On the other hand, the amide and ester groups in PABS can form strong hydrogen bonds with hydroxyl groups in PVA, generating the non-covalent bonding within the gel, which can be easily broken upon the addition of acid, leading to superior acid degradability.

## 2. Results and Discussion

### 2.1. Chemical Structure

**Figure 1:**
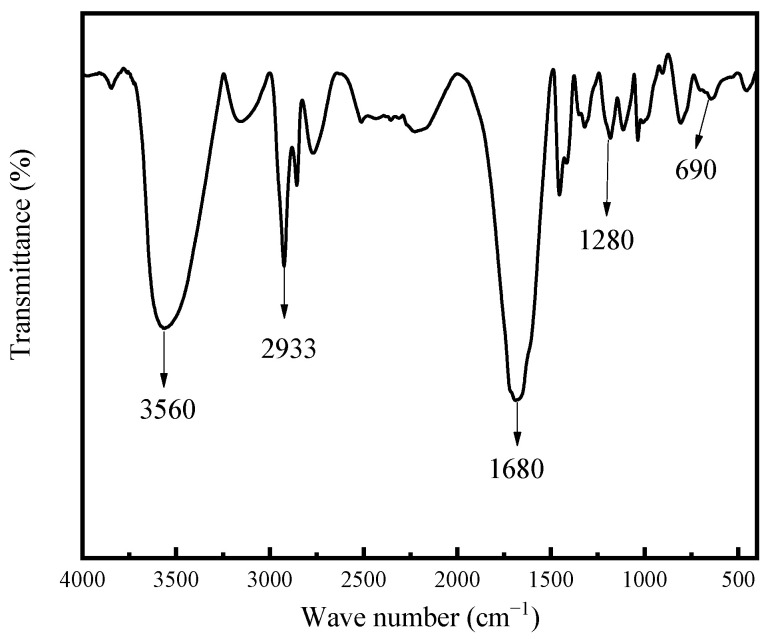
Fourier transform infrared spectrum of supramolecular polymer gel.

The molecular structure of the supramolecular gel was characterized using Fourier transform infrared spectrum (Figure 1). The characterization results show that the peak at 690 cm^−1^ is the characteristic peak of benzene ring bending vibration, indicating the presence of ST structure in the sample. The peak at 1280 cm^−1^ is due to the C-N contraction vibration from AM [34]. The peak at 1680 cm^−1^ corresponds to the amide group, and the peak at 2933 cm^−1^ corresponds to the CH_2_ asymmetric contraction vibration [35]. In addition, the wide and strong peak at CH_2_ 3300–3650 cm^−1^ is mainly attributed to the hydroxyl group of PVA as well as the formation of hydrogen bonding interaction between PVA and AM or BA [36,37,38]. The benzene ring, amide group, hydroxyl group, and other functional groups present in the gel sample confirmed the formation of supramolecular polymer gel based on PABS and PVA.

### 2.2. Thermal Properties

**Figure 2:**
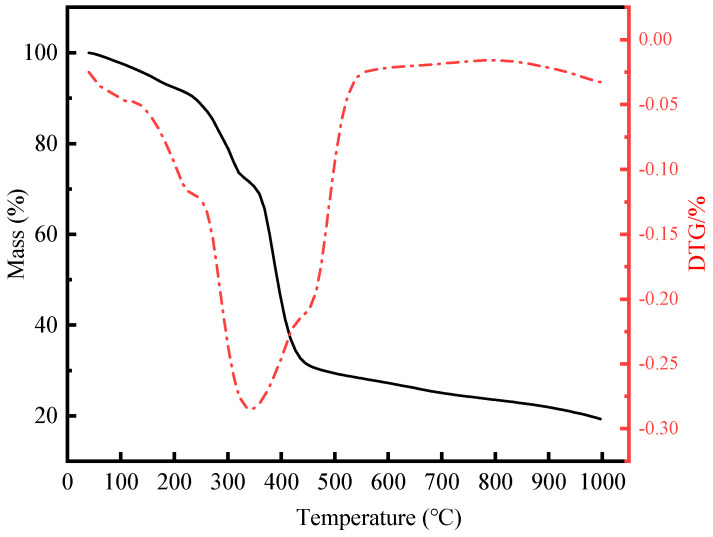
Thermogravimetric analysis of supramolecular polymer gels.

Thermogravimetric analysis shows that the first stage of gel sample weight loss occurs at 30–250 °C, during which the mass of the gel sample decreases slightly with the increase in temperature (Figure 2). The main reason for this decrease in mass is the evaporation of a small amount of free and bound water molecules in the supramolecular polymer gel sample [39,40]. The second weight-loss stage took place between about 250–430 °C, during which the mass loss rate of the gel sample was extremely high, indicating that the molecular structure of the supramolecular polymer gel system began to break down and the intermolecular covalent bonds were broken. In the final stage, when the temperature exceeded 430 °C, the mass loss rate gradually stabilized, and the network structure of the supramolecular polymer gel system basically collapsed. When the temperature reached 1000 °C, the final residual mass was about 15.9%.

### 2.3. Morphology

**Figure 3:**
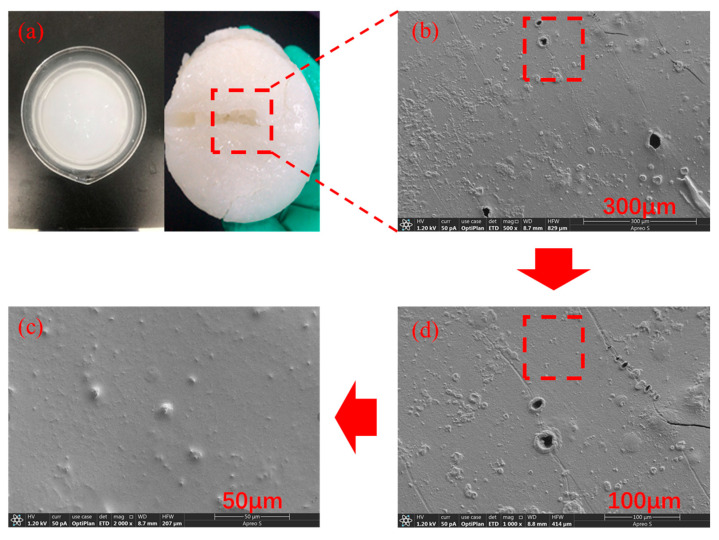
Morphology of supramolecular polymer gel. (**a**) Macroscopic picture of supramolecular polymer gel; (**b**) Micromorphology of supramolecular polymer gel at 300 μm; (**c**) Micromorphology of supramolecular polymer gel at 50 μm; (**d**) Micromorphology of supramolecular polymer gel at 100 μm.

The surface morphology of the supramolecular polymer gel was analyzed by electron microscopy (Figure 3). At a scale of 300 μm, the surface of the supramolecular polymer gel showed a uniform and smooth morphology, with almost no significant rough regions or irregular prominences [41]. At a scale of 100 μm, the smooth morphology, absence of obvious fractures, and presence of only a few microporous structures indicated that the gel underwent sufficient self-assembly and crosslinking reactions during the preparation process, resulting in compact films. Finally, at a scale of 50 μm, the smooth morphology of the gel surface was more evident. This smoothness and compactness across multiple scales indicated that the supramolecular polymer gel has an excellent microstructure that resists external forces, which facilitates its superior plugging performance.

### 2.4. Mechanical Performance

**Figure 4:**
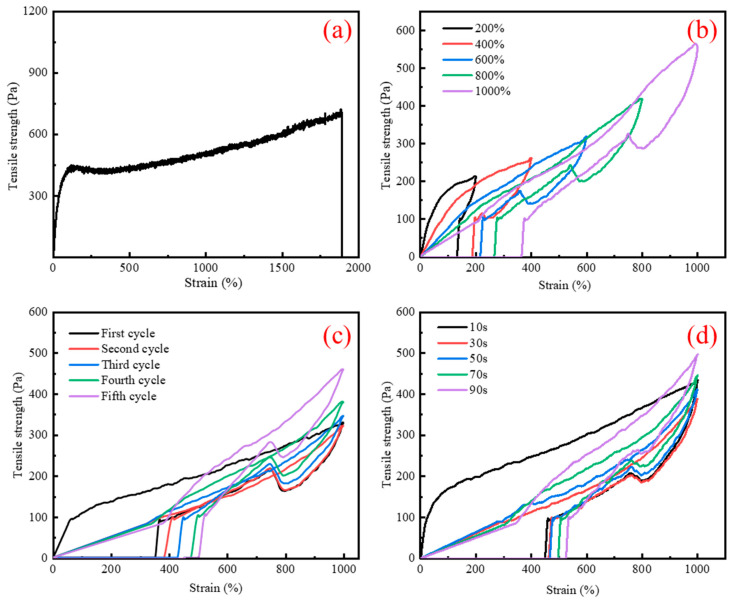
Mechanical performance of supramolecular polymer gel: (**a**) tensile stress–strain curves; (**b**) cyclic tensile curve with variable strains; (**c**) cyclic stretching curve with variable cycles; (**d**) cyclic stretching curve with variable times.

As shown in Figure 4a, the supramolecular polymer gel exhibited a maximum tensile elongation of 1800% and a maximum fracture stress of 650 Pa. Compared with the supramolecular polymer gel reported previously [42], the supramolecular polymer gel has higher tensile strength and elongation. Supramolecular polymer gel samples were subjected to continuous tensile tests at fixed strains of 200%, 400%, 600%, 800%, and 1000%. As shown in Figure 4b, at the initial 200% strain, the tensile strength was about 200 Pa. After stretching at different strains, the supramolecular polymer gel still had excellent elasticity and strength. Then, a fixed strain of 1000% was applied to the sample over five cycles with no time interval, and its recovery efficiency was recorded, as shown in Figure 4c. At the initial cycle, the maximum strength was about 300 Pa, and after multiple cycles, the tensile strength remained stable with little change in the cycle curve, which highlights the excellent fatigue resistance and recovery properties of the gel. Finally, under a fixed strain of 1000%, the supramolecular polymer gel sample was tested for five cycles at different time intervals (Figure 4d) [43,44]. It was observed that supramolecular polymer gels had better fatigue resistance and recovery properties compared to continuous stretching without intervals (Figure 4c). MBA serves as a crosslinking agent in this system, capable of forming chemical crosslinking points between polymer chains. These crosslinking points limit the relative movement of the polymer chain, so the mechanical strength of the gel was improved. In addition, BA can endow gel with suitable toughness and ductility, while ST can improve the strength and modulus of gel. The appropriate combination of the two was the key to achieving high-strength tensile performance.

### 2.5. Influence of Saline Water Content on the Gelation and Rheology of Resultant Gels

The heavy oil reservoir contains a large amount of mineral water. When the gel-forming solution encounters the mineral water in the reservoir, it can be diluted and contaminated by salts. As a consequence, the formation of supramolecular polymer gel might be affected, leading to a poor sealing effect. Therefore, it is necessary to explore the effect of saline water content on the gelation of the gel-forming solution.

**Figure 5:**
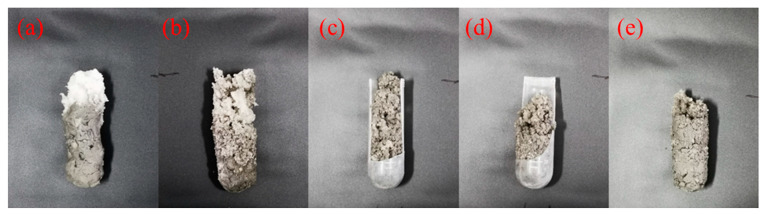
Digital images of gels formed after mixing gel-forming solution with saline water at different volume ratios: (**a**) 1/5, (**b**) 2/5, (**c**) 3/5, (**d**) 4/5, and (**e**) 5/5.

**Figure 6:**
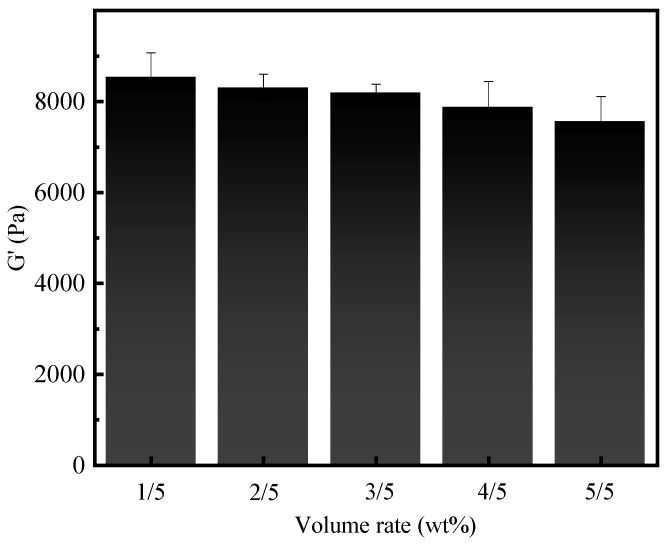
Storage modulus of gels formed after mixing gel-forming solution with saline water at different volume ratios.

In this study, saline water with 150,000 mg·L^−1^ NaCl was prepared and mixed with the gel-forming solution at different volume ratios of 1/5, 2/5, 3/5, 4/5, and 5/5 (saline water/gel-forming solution). These mixtures were then poured into a centrifuge tube and placed in a high-temperature environment at 120 °C to form the gels. As shown in Figure 5, supramolecular polymer gel can form a gel at the whole volume ratios investigated, indicating that it has suitable resistance to salt water dilution and salt pollution [17,45]. Furthermore, we evaluated the viscoelasticity of resultant gels using a Harker rheometer. As shown in Figure 6, the storage modulus of resultant gels gradually decreased from 8534 to 8303, 8190, 7872, and 7555 Pa with an increase in the volume ratios from 1/5 to 2/5, 3/5, 4/5, and 5/5. At the highest ratio of 5/5, the storage modulus was still as high as 7555 Pa. These experimental results strongly demonstrated that the supramolecular polymer gel-forming system still maintained superior gelation capacity in a high-brine environment, and the resultant gels exhibited high storage modulus, showing its excellent resistance to brine invasion [46].

### 2.6. Influence of Heavy Oil Content on the Gelation and Rheology of Resultant Gels

**Figure 7:**
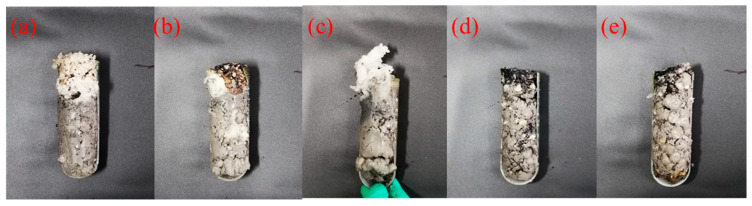
Digital images of gels formed after mixing gel-forming solution with heavy oil at different volume ratios: (**a**) 2/98, (**b**) 4/96, (**c**) 6/94, (**d**) 8/92, and (**e**) 10/90.

**Figure 8:**
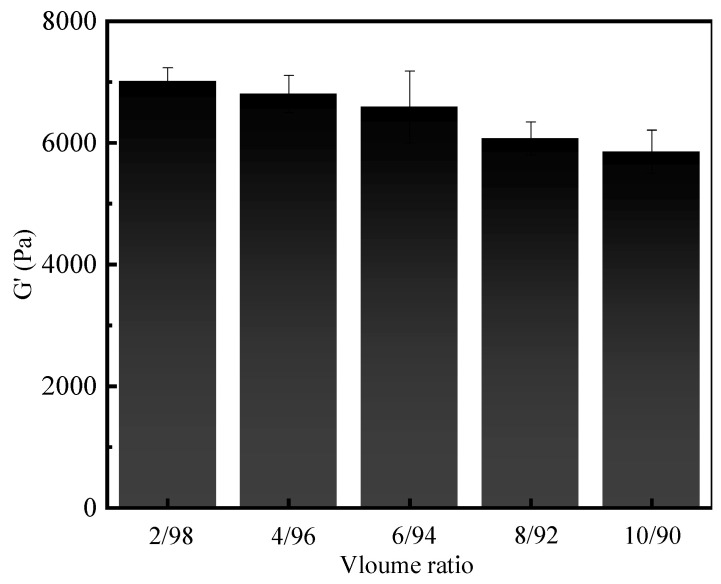
Storage modulus of gels formed after mixing gel-forming solution with heavy oil at different volume ratios.

In addition to mineral water, the reservoir contains abundant heavy oil. The heavy oil can penetrate into the wellbore along the fractures, which might affect the gelation process, as well. In this study, the influence of heavy oil content on the gelation of gel-forming solution was thereby studied. The gel formation test was carried out by mixing heavy oil and gel-forming solution in different volume ratios of 2/98, 4/96, 6/94, 8/92, and 10/90. As shown in Figure 7, after mixing different volumes of heavy oil, all systems can also form gels, exhibiting excellent resistance to heavy oil and pollution. Additionally, the viscoelasticity of resultant gels was evaluated. As shown in Figure 8, the storage modulus of resultant gels gradually decreased from 7012 to 6803, 6590, 6072, and 5855 Pa with an increase in the volume ratios from 2/98 to 4/96, 6/94, 8/92, and 10/90. At the highest ratio of 10/90, the storage modulus was still as high as 5855 Pa. Therefore, the supramolecular polymer gel-forming system possessed superior resistance to heavy oil contamination [47,48,49].

### 2.7. Fracture Sealing Performance

**Figure 9:**
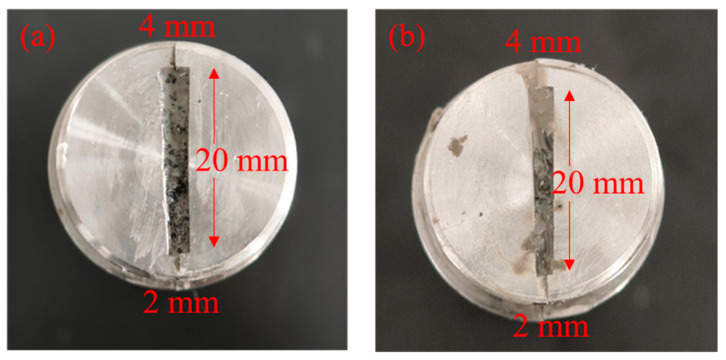
The digital images of wedge-shaped fracture: (**a**) inlet and (**b**) outlet.

The superior mechanical properties of supramolecular polymer gels, along with excellent resistance to saline water and heavy oil contamination of the gel-forming solution, ensured their effectiveness in sealing fractures to prevent the intrusion of heavy oil from the reservoir into the wellbore. To study the fracture sealing performance, the gel-forming solution was injected into a fracture core of wedge-shaped steel columns (Figure 9) with a length of 30 cm at an injection speed of 9.99 mL/min in a high-temperature and high-pressure plugging device. The temperature was then increased to 120 °C to initiate the reaction. After gelation, the gel was displaced by water to evaluate the plugging effect.

**Figure 10:**
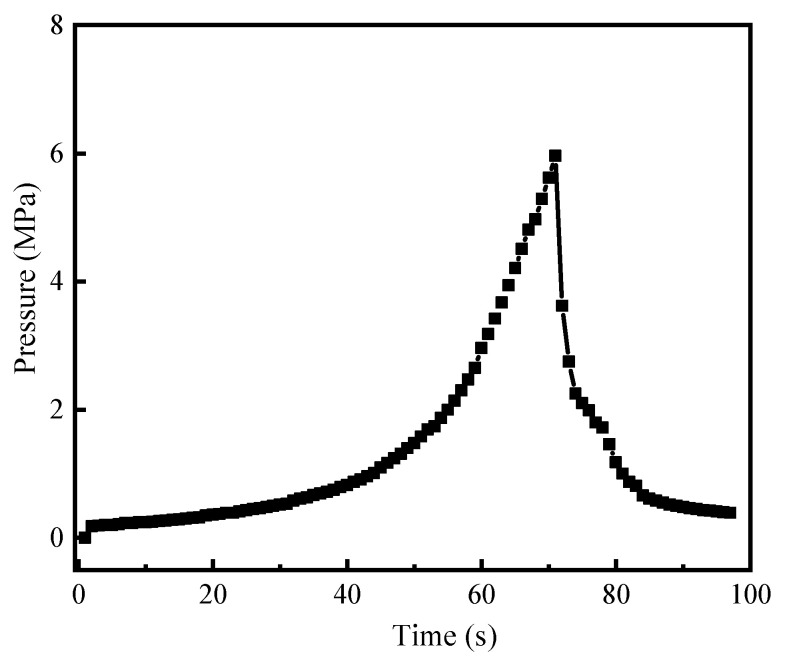
Curve of pressure strength over time.

As shown in Figure 10, with the continuous injection of fluid at the rate of 9.99 mL/min, the fluid pressure on the gel in the wedge fracture gradually increased [50,51,52]. With the progress of polymerization, monomer molecules are gradually transformed into polymer chains, and network structure is formed under the action of a crosslinking agent. This network structure has suitable water swelling and viscoelasticity. The gel can closely fill fractures, and effective physical barriers are formed. When it reached 5.96 MPa, the pressure dropped instantaneously, indicating that the gel integrity was destroyed under the action of fluid pressure, indicating that the maximum breakthrough pressure of gels in the wedge fracture was 5.96 MPa. The inward fluid flowed out of the core outlet, and thereby, the pressure dropped gradually. Compared with other temporary plugging systems, e.g., preformed gel, polyethylene mine/partially hydrogenated polyacrylamide gel, and β—cyclodextrin-based supramolecular polymer gel [30,34,53], the gel system demonstrated better fracture plugging performance and pressure endurance.

### 2.8. Acid Solubility

For temporary plugging fractures in heavy oil reservoirs, the acid solubility of supramolecular polymer gels is also very important. If gel cannot be degraded after the plugging purpose is served, it may cause serious damage to the reservoir and affect the subsequent heavy oil recovery. Therefore, the acid solubility of supramolecular polymer gel was evaluated [54].

**Figure 11:**
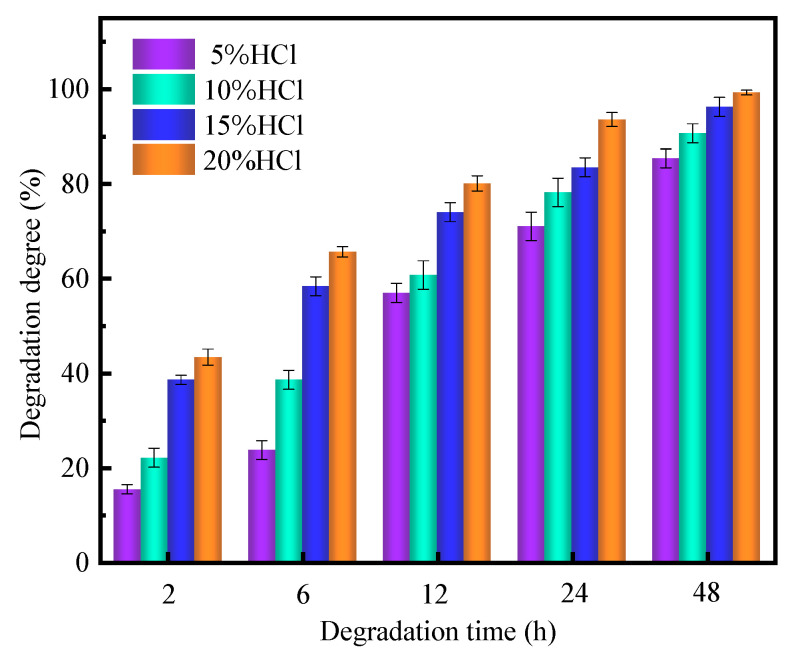
Degradation degree of gel at different times and concentrations of HCl.

As shown in Figure 11, when the concentration of HCl was 5%, the dissolution rate reached 85.3% after 48 h. In the 10%, 15% HCl, and 20% HCl environments, the dissolution rate reached 90.6%, 96.2%, and 99.2% after 48 h, respectively. The dissolution rate increased with an increase in acid concentration. In addition, at the same HCl concentration, the dissolution rate also gradually increased with an increase in treatment time. The reason why the degradation degree of gel increases is that with the increase in hydrochloric acid concentration, the concentration of hydrogen ions (H^+^) in the solution increases accordingly. These hydrogen ions can interact more frequently with the chemical bonds in the gel (i.e., amide bonds, hydrogen bonds, etc.), accelerate the breaking of chemical bonds, and thus accelerate the degradation rate of the gel. The results of acid dissolution experiments showed that the supramolecular polymer gel system exhibited suitable acid degradation performance, meeting the temporary plugging needs [23,55]. During the drilling process, the gel-forming solution can be added and then initiated to form robust supramolecular polymer gels in the fractures, avoiding the leakage of heavy oils from the reservoir into the wellbore. When heavy oils need to be extracted after drilling, an HCl solution can be used to dissolve the supramolecular gel in the fracture, leading to enhanced heavy oil productivity.

## 3. Conclusions

To solve the problem of heavy oil intrusion into the wellbore, a supramolecular polymer gel with high strength and degradability was prepared based on radical polymerization of acrylamide, butyl acrylate, and styrene monomers, using N, N-methylene bisacrylamide as a crosslinker, ammonium persulfate as an initiator, and polyvinyl alcohol as a non-covalent component. According to our study, the following conclusions can be made:

(1) The infrared spectrum results showed that the gel contains the characteristic functional groups of polyvinyl alcohol, acrylamide, butyl acrylate, and styrene, proving that the supramolecular gel was successfully synthesized. The gel surface is smooth and flat, which greatly improves the strength of the supramolecular polymer gel system;
(2) The supramolecular polymer gel exhibited suitable mechanical properties. The maximum elongation at the break of the gel was 1800%, and the maximum tensile strength was 650 Pa;
(3) The supramolecular polymer gel showed suitable contamination resistance toward brine and heavy oil, and its strength reached 7555 Pa and 5855 Pa even after mixing with brine (50 vol%) and heavy oil (10 vol%), respectively;
(4) For the wedge-shaped fracture (20 mm × 4 mm and 20 mm × 2 mm), the breakthrough pressure of the gel reached 5.96 MPa, demonstrating the excellent sealing properties of the supramolecular polymer gel;
(5) After adding a 15% HCl solution for 48 h at 120 °C, the gel dissolution rate reached 96.2%, indicating that the supramolecular polymer gel system possessed suitable degradation performance, which was conducive to subsequent plugging removal and heavy oil recovery.

Overall, with its superior mechanical strength, saltwater and heavy oil resistance, pressure-bearing capacity, and acid degradability, the supramolecular polymer gel-based temporary plugging agent shows promising applications in heavy oil reservoirs to prevent the intrusion of heavy oil while maintaining high productivity.

## 4. Materials and Methods

### 4.1. Materials

Acrylamide (AM) was purchased from Tianjin in China Ruijinte Chemical Co., Ltd. (Tianjin, China). Butyl acrylate (BA) and styrene (ST) were purchased from Shanghai in China McLean Biotechnology Co., Ltd. (Shanghai, China). N, N-methylene bisacrylamide (MBA), polyvinyl alcohol (PVA), and ammonium persulfate (APS) were purchased from Sinopharm Group Chemical Reagents Co., Ltd. (Shanghai, China).

### 4.2. Preparation of Supramolecular Polymer Gels

First, 10 wt% PVA solution was prepared by adding 10 g PVA particles in 90 mL water at 95 °C, followed by mechanical stirring until complete dissolution. Then, 20 g AM, 3 mL BA, 3 g ST, and 0.2 g MBA were added to the above PVA solution. The mixture was heated at 120 °C, followed by purging nitrogen for 30 min to remove oxygen. Then, 0.2 g APS was gradually added to initiate the reaction. The reaction was conducted for 6 h to obtain supramolecular polymer gel.

### 4.3. Fourier Transform Infrared Spectroscopic Analysis

The chemical structure of the gel was characterized by Fourier transform infrared spectroscopy (IRSpirit, Tokyo, Japan). The prepared supramolecular polymer gel was dried, crushed into powder, and then mixed with pure potassium bromide, followed by pressing to obtain the sample. The test range was 500–4000 cm^−1^ with a resolution of 2 cm^−1^.

### 4.4. Thermogravimetric Analysis

The thermal stability of the gel was studied using a TGA400 thermogravimetric instrument (METTLER TOLEDO, Phoenix, AZ, USA). The heating temperature is 40~1000 °C, and the heating rate is 5 °C/min, with N_2_ protection.

### 4.5. Micromorphology Analysis

The surface morphology was observed by Sigma 360 scanning electron microscope (Zeiss, Berlin, Germany). Prior to observation, the surface of the sample was coated with a layer of gold by spraying.

### 4.6. Mechanical Performance Testing

The mechanical properties of the samples were tested by an electronic universal testing machine (HD-B6115-s, Suzhou, China). The size of the gel sample was 4.5 cm × 0.2 cm × 0.2 cm, and the tensile rate was fixed at 100 mm/min.

### 4.7. Rheological Analysis

The viscoelasticity of the gel was characterized by a MARS60 rheometer (HAAKE, Berlin, Germany). The frequency sweep was conducted at a constant strain of 10% in the frequency range of 0.1~100 rad/s at 25 °C.

### 4.8. Plugging Performance Analysis

The plugging performance of supramolecular polymer gel was tested by using a high-temperature and high-pressure plugging device. The precursor solution of supramolecular polymer gel was injected into the fracture core model of the steel column. After 6 h of complete reaction at 120 °C, clean water was injected. The pressures at the outlet during water injection were recorded.

### 4.9. Acid Degradation Measurement

Supramolecular polymer gel sheets with equal thickness were prepared and pressed into gel blocks of the same size and quality using precision molds. Then, HCl solutions with different concentrations of 5, 10, and 15 wt% were prepared. To simulate the temperature conditions of the heavy oil reservoir (120 °C), these gel samples were placed in the HCl solution under 120 °C. After a preset interval, the gel blocks were removed and dried, and their mass was measured to calculate the dissolution rate of the gel accordingly.

## Data Availability

The data presented in this study are openly available in article. Informed consent was obtained from all subjects involved in the study.

## Abbreviations

Amino gelatin (AG), ammonium persulfate (APS), acrylamide (AM), butyl acrylate (BA), polylactic acid (PLA), poly (acrylamide-co-butyl acrylate-co-styrene) (PABS), polyvinyl alcohol (PVA), styrene (ST), sodium aldehyde alginate (ASA), N, N-methylene bisacrylamide (MBA).
